# A step beyond in steady-state and time-resolved electro-optical spectroscopy: Demonstration of a customized simple, compact, low-cost, fiber-based interferometer system

**DOI:** 10.1063/4.0000134

**Published:** 2022-01-13

**Authors:** Giovanni Pica, Daniele Bajoni, Giulia Grancini

**Affiliations:** 1 Department of Chemistry and INSTM, University of Pavia, Via T. Taramelli 14, 27100 Pavia, Italy; 2 Department of Electrical, Computer and Biomedical Engineering, University of Pavia, Via A. Ferrata 1, 27100 Pavia, Italy

## Abstract

Electro-optical spectroscopy is nowadays a routine approach for the analysis of light induced properties and dynamical processes in matter, whose understanding is particularly crucial for the intelligent design of novel synthetic materials and the engineering and optimization of high-impact optoelectronic devices. Currently, within this field, it is the common choice to rely on multiple commercial setups, often costly and complex, which can rarely combine multiple functions at the same time with the required sensitivity, resolution, and spectral tunability (in both excitation and detection). Here, we present an innovative, compact, and low-cost system based on “three in one” components for the simultaneous electro-optical material and device characterization. It relies on compact fiber-coupled Fourier transform spectroscopy, the core of the system, enabling a fast spectral analysis to acquire simultaneously wavelength and time resolved photoluminescence (PL) maps (as a function of the time and wavelength), PL quantum yield, and electroluminescence signal. Our system bypasses conventional ones, proposing a new solution for a compact, low-cost, and user-friendly tool, while maintaining high levels of resolution and sensitivity.

## I. INTRODUCTION

Electro-optical measurements including standard photoluminescence (PL), both wavelength and time resolved analysis (TRPL), transmittance, PL quantum yield (PLQY), or PL excitation spectra, to name a few, are nowadays common tools for the optoelectronic characterization of light-induced physical-chemical properties in a wide range of materials, including natural biomaterials, artificial inorganic systems, and organic molecules, but also to directly test the working principle, potential losses, or new phenomena directly on working devices.^1–7^ This is the case, for instance, of optoelectronic devices, such as solar cells, light emitting devices, and sensors, where this information enables one to directly target not only the material properties but also, importantly, the device processes, such as the radiative and non-radiative decay channels, losses, and charge transport mechanisms, providing the instrument for the real understanding of the device physics and performances.^6,8–11^ Indeed, the photoluminescence lifetime is an intrinsic characteristic of a luminescent species that can provide insights into the species excited state dynamics. Time-resolved photoluminescence is the tool of choice for studying fast electronic deactivation processes that result in the emission of photons, a process called fluorescence. Fluorescence decays take place on the pico/nanosecond timescale, and they can be influenced by environmental processes occurring on these ranges, meaning that they are an ideal instrument to track an emissive activation path in molecules and semiconductors, which usually happen in a range from few picoseconds to hundreds of nanoseconds. Therefore, fluorescence spectroscopy is at the same time a qualitative probe to investigate the nature of photoexcited species (molecular states or carrier dynamics in semiconductors) as well as a quantitative indicator of the radiative vs non-radiative paths, which strongly depend on molecular interactions, electron–phonon coupling, and the structural order of the material.^9,11^ In this work, we used a study case system of a semiconductor in the form of methylammonium lead iodide hybrid perovskites (MAPbI_3_), where the photoluminescence signal probes the diffusion of the photoexcited carriers happening over tens of nanoseconds, from which a diffusion length of around 100 nm has been estimated.^12^ In addition, the PL quantum yield quantifies the non-radiative losses usually related to bulk and surface defects in the materials.^10^ In conventional commercial setups, the spectroscopic system is usually composed of three main blocks: (i) the excitation light source, (ii) the spectral- and/or time-resolving analyzer, and (iii) the detection system, which can be coupled with additional elements (such as an integrating sphere or a source meter) allowing for simultaneous spectral and temporal analysis.^6^ For instance, time-resolved photoluminescence today has become a highly needed setup, which is commonly available in research and industrial laboratories. The most sophisticated—and expensive—systems, asking for high temporal resolution and sensitivity, incorporate components, such as upconversion systems, that allow time resolution below hundreds of femtoseconds, despite reduced sensitivity, or streak cameras with a time resolution of the order of picoseconds and sensitivity down to the single photon level, despite cost and frailty.^13–15^ However, the use of a streak camera has many drawbacks in terms of cost and complexity of the system, being based on the use of a spectrograph. Alternatively, time correlated single photon counting (TCSPC) systems offer a good compromise, combining good temporal resolution (down to a few tens of picoseconds) together with robustness and single photon sensitivity. Current commercial equipment involves, therefore, rather complex systems based on a serial approach, in which a monochromator is usually placed in front of the detector, and fluorescence decay is recorded wavelength by wavelength, building the spectral and time resolved map step-by-step with long (hours) time consumption. Notably, in these cases, spectral analysis is done using single or double monochromator stages depending on the needed spectral resolution.^16^ Typically, light is focused on the monochromator input slit and diffracted by sequential gratings or diffraction elements and collected sequentially by rotating the grating and then selecting the wavelength. Double monochromators are usually used to enhance the spectral accuracy at the cost of reduced sensitivity. It is clear that, in the case of low-light applications, e.g., fluorescence, the monochromator throughput can often be too small to achieve sufficient signal to noise ratio values (typically around 10^2^) during the integration times compatible with the experiments. In this sense, the use of monochromators followed by single photon detectors (SPDs) introduces a trade-off between spectral resolution and signal to noise ratios in the time resolved histograms. In addition, common gratings have a limited efficiency and a narrow spectral bandwidth, resulting in a reduced spectral coverage. The use of CCD cameras for the detection of a broad spectral range represents an alternative approach that can overcome the problems of low sensitivity and long acquisition time but at the expense of their price. Overall, this results in an increased complexity and cost of the whole system and long and time-consuming acquisitions.

In this work, we designed an innovative method for electro-optical measurements, which surpasses such limitations providing a compact and easy to implement characterization system while keeping high spectral sensitivity and time resolution. This has been obtained by substituting the spectrophotometer unit with a compact, fiber-coupled Fourier transform interferometer.^17,18^ The advantages include easier alignment (no input or output slits) and light coupling, higher throughput (no needs for filters), flexibility, compactness, and robustness. In addition, a broader spectral range is ensured (no gratings) from 250 to 1200 nm without the need of changing diffraction elements, making this configuration a versatile, compact, and innovative way to perform standard as well as more advanced electro-optical measurements.

## II. EXPERIMENTAL SETUP: DESIGN

**FIG. 1.**
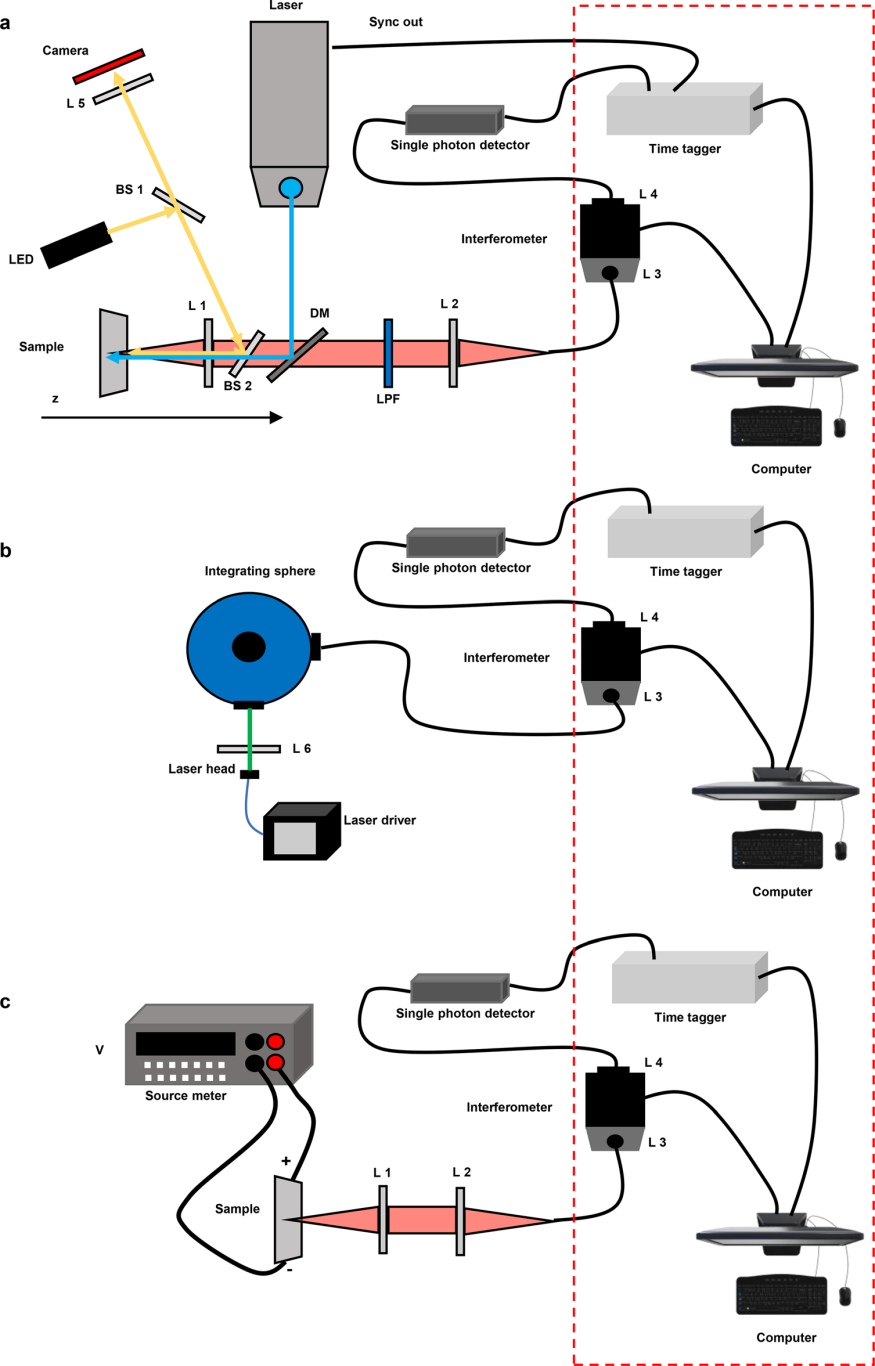
Schematic representation of the experimental setup for (a) photoluminescence and time-resolved photoluminescence, in which L, BS, DM, and LPF refer to lens, beam splitter, dichroic mirror, and long pass filter, respectively, (b) photoluminescence quantum yield, and (c) electroluminescence analysis. Red dashed square indicates that the detection part of the system remains unchanged throughout all the measurements.

A schematic diagram of the experimental setup is shown in Fig. 1.

**FIG. 2.**
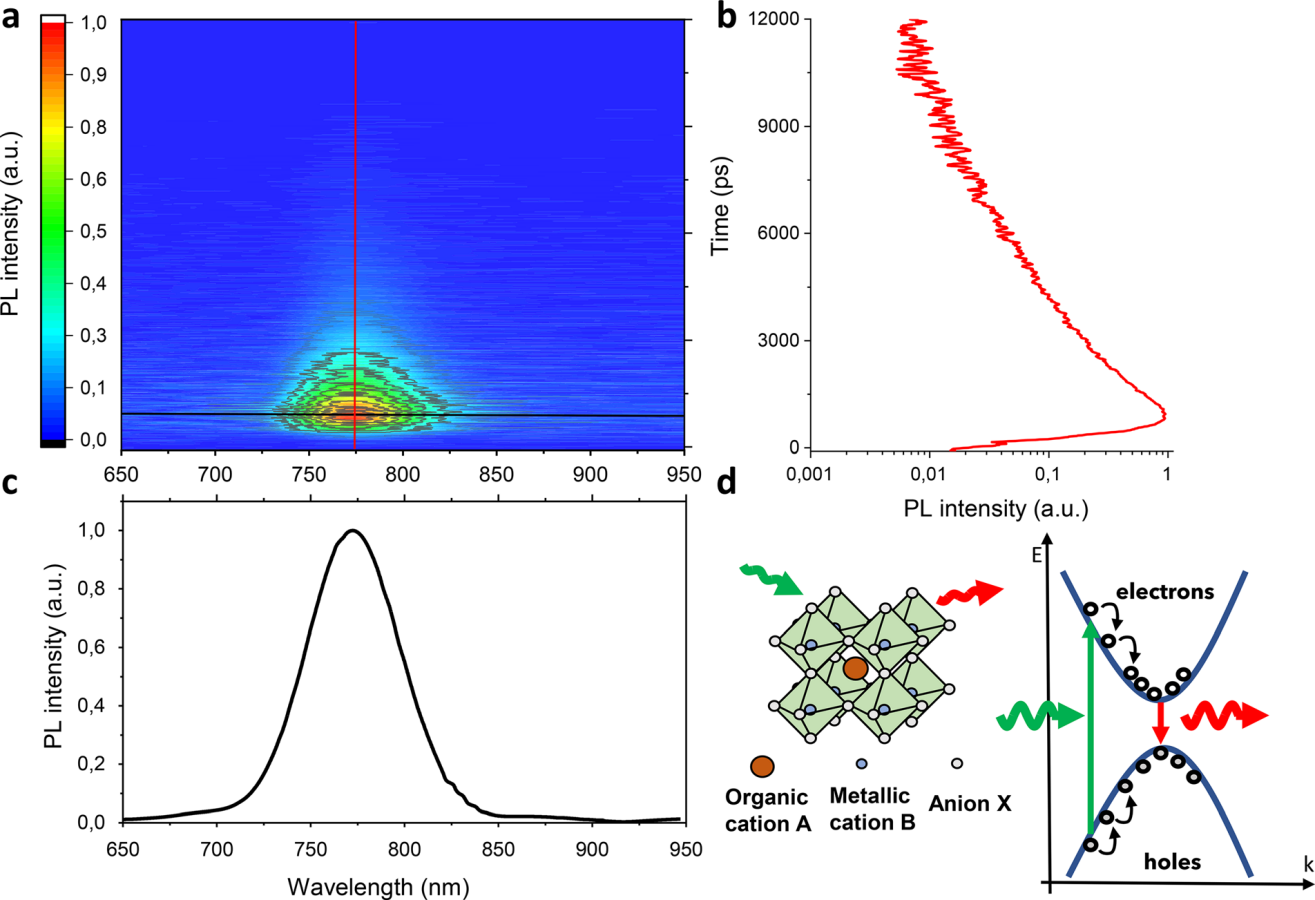
Example of wavelength and time resolved photoluminescence measurements on a hybrid perovskite solar cell. Time and wavelength resolved map (a) allows the simultaneous analysis of the photoluminescence emission as a function of time (b) and wavelength (c). Typical structure of a hybrid perovskite and schematic representation of the photoluminescence mechanism.

It consists of a three in one system for simultaneous detection of the (1) PL signal (spectra and time resolved with a single photon counting unit)—Fig. 1(a); (2) PLQY with the integrating sphere—Fig. 1(b); and (3) electroluminescence (EL), where the samples are biased with a source meter—Fig. 1(c). The core of the setup is the interferometer, coupled with a single photon detector and the time tagger. In brief, the interferometer (GEMINI, by NIREOS) consists of two polarizers oriented at 45° and two birefringent optical elements with crossed optical axis and perpendicular to the propagation direction of the beam. Due to the presence of the first polarizer, the light results in the superposition of two replicas with perpendicular polarization. The birefringent elements introduce a tunable delay (due to the tunable thickness of one of the two blocks of the birefringent material) between the replicas that can interfere constructively or destructively after passing through the second polarizer that projects the two replicas onto the same polarization state to ensure their interference at the detector (see the supplementary material for additional details). The geometry of the birefringent elements is such that the maximum delay between the two replicas is around 2.1 ps. This delay provides a maximum spectral resolution of 0.57 nm at λ = 600 nm wavelength, much higher than that required to resolve the typical emission linewidths of fluorescent samples at room temperature. The system here used is to extend the principle of Fourier transform spectroscopy for the realization of our compact multiple electro-optical setup, described below in more detail.

### A. PL and time resolved PL (TRPL) setup

For the PL and the TRPL, a dual mode [continuous wave (CW) and pulsed] laser at 470 nm with tunable repetition rate (from single shot to 40 MHz) and integrated collimators is used as the excitation source (PicoQuant, LDH series D-C-470). The parameters of the laser used can affect the quality of the measurements in terms of resolution and/or signal to noise ratios, meaning that the choice of the source has to be carefully considered when designing the whole system. In more detail, the laser adopted in the setup shown has a pulse width of 64 ps with a peak power of 221 mW and a pulse energy of 21 pJ at 40 MHz, showing a bandwidth of 6 nm and an instrumental response function (IRF) of 30 ps. The excitation beam [blue line in Fig. 1(a)] is reflected by a long-pass dichroic mirror at 500 nm and passes through a focusing lens (L 1 in Fig. 1). To facilitate proper alignment and focusing, the target sample is placed on a micrometric stage that can be finely adjusted on the z-axis. The PL signal is collected back in the epifluorescence mode by L 1 transmitted by the dichroic mirror. Then, in order to remove any stray light, an additional long pass filter is placed in front of L 2, which focuses the beam into an optical fiber connected to the interferometer. This leads to a compact and robust alignment avoiding free space light and simultaneously leading to a safer and easily aligned system. The output of the interferometer is connected through an optical fiber to the single photon detector (SPD hereafter). A time tagger collects the signal from the SPD and the synchronization signal from the laser, enabling time correlated single photon counting (TCSPC) experiments with a time resolution of tens/hundreds of picoseconds depending on the specific SPD and time tagger used. The interferometer and time tagger are connected via USB (universal serial bus) to a computer for software analysis and data collection. As we will discuss later, the result of this measurement is a two-dimensional (2D) map of the fluorescence signal as a function of the emission wavelength and decay time. By judicious selection, it is possible to retrieve the PL signal at the selected time frame with a sensitivity of 1 nm on the central wavelength of the range spanned.

### B. PLQY measurements

**FIG. 3.**
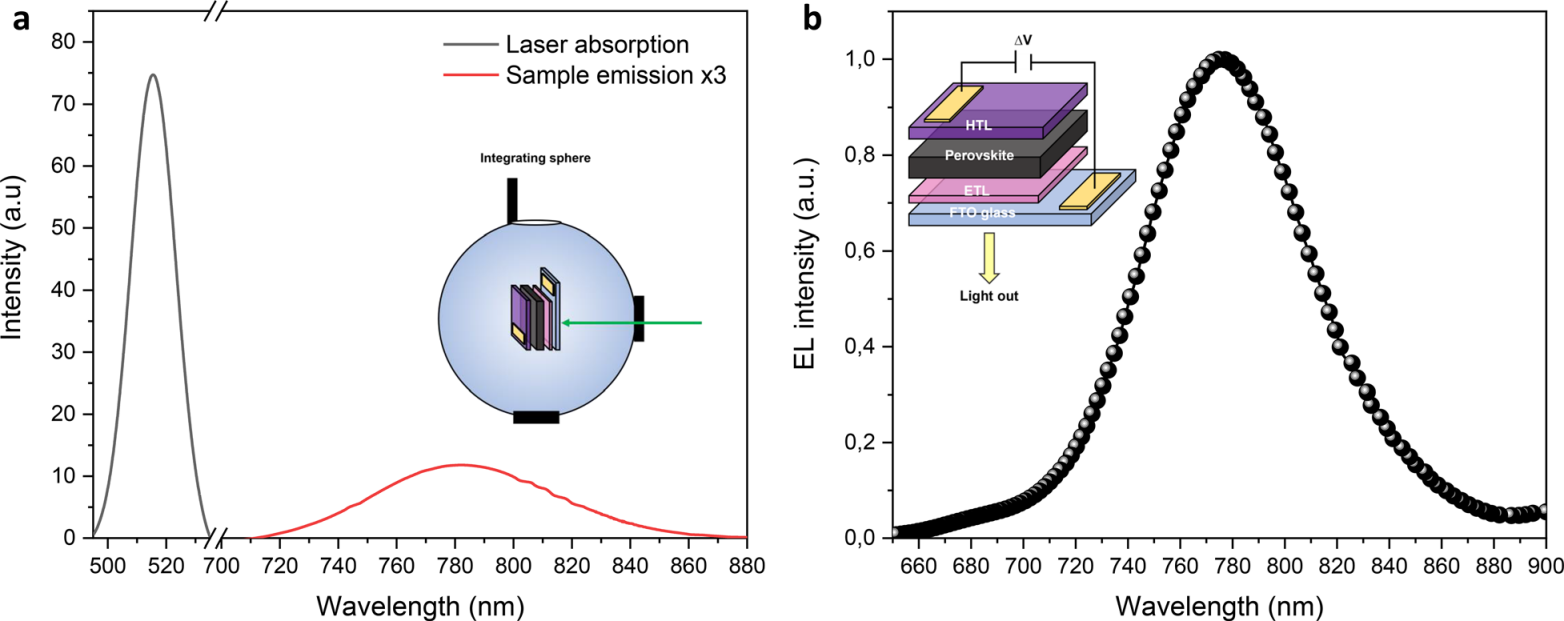
Examples of the photoluminescence quantum yield (a) and electroluminescence (b) measurements.

The system above represents the core of a versatile tool, which allows for the simultaneous measurement of multiple parameters. Among others, the quantitative analysis of the PLQY is pivotal for the estimation of the radiative vs non-radiative losses, which is of fundamental importance for the understanding material and device physics. The PLQY setup is shown in Fig. 1(b). The detection part of the setup is analogous to the one mentioned above, being constituted by the SPD coupled with the time tagger. However, in this case, the fiber entering the interferometer is connected to a 6-in. integrating sphere (LabSphere, CSTM-QE-060-SF). The laser source is focused through L 6 on the sample placed inside the sphere [the inset in Fig. 3(a)]. A customized software is developed for the correct analysis of the spectra collected and for the calculation of the PLQY value.^1^ Notably, such measurements can be performed among thin films, solutions, or directly on a device. As in the case of the PL-TRPL map, the sensitivity of the system is related to the detector used. The minimum signal recorded is of 10^−4^.

### C. Electro-luminescence (EL) measurements

In order to further supplement the optical characterization, electric analyses allow for testing the radiative and non-radiative losses directly into a device. Figure 1(c) shows the experimental configuration of the EL. In this case, a source meter sources a voltage across the sample. The injected carriers recombine radiatively, and the light emitted is collected through the same detection system mentioned above, constituted by an interferometer and an SPD connected to a time tagger.

## III. EXPERIMENTAL PROOFS OF CONCEPT ON HYBRID PEROVSKITE THIN FILMS AND HIGHLY EFFICIENT SOLAR CELLS

We performed the above mentioned measurements on the *ad hoc* fabricated methylammonium lead iodide hybrid perovskite [MaPbI_3_, whose structure is shown in Fig. 2(d)] film and MaPbI_3_-based solar cell.^3,19^ MaPbI_3_ films have been fabricated by standard spin coating techniques, resulting in a 200 nm thick film, subsequently incorporated within an electron/hole transporting layers' (ETL and HTL, respectively) stack in a full device configuration [the inset in Fig. 3(b)].^20,21^

Figure 2 shows an example of the wavelength and time resolved PL measurement on a hybrid perovskite thin film.

The luminescence emitted by the sample is collected by the SPD (IDQuantique, ID100) and connected to the time tagger (Swabian Instrument, TimeTagger Ultra), providing the photon time-of-flight distribution on a temporal window of 25 ns. The photon count-rate is fixed at 1 MHz to fulfill the statistical limit of the TCSPC technique (1%–5% of the pulse repetition rate).^22^ This, in turn, sets the integration time to 0.1 s, which is required to obtain a sufficient signal-to-noise ratio (SNR) in the histogram of the fluorescence decay at a given position of the birefringent interferometer. The instrumental response function (IRF) of the detection chain has a FWHM of 40 ps. Figure 2(a) shows a time and wavelength resolved map that allows the simultaneous visualization of the emission as a function of both time and wavelength. It is possible to disentangle the two different investigations by analyzing single cuts along the map. Additionally, by performing a vertical cut [red line in Fig. 2(a)], i.e., by fixing the emission wavelength, it is possible to follow the temporal decay of the luminescence at that specific wavelength, shown in Fig. 2(b). Conversely, by performing a horizontal cut [black line in Fig. 2(a)], i.e., by fixing the time, it is possible to retrieve the emission spectrum as a function of the wavelength, shown in Fig. 2(c). These kinds of measurements are usually employed to verify the correct crystalline structure of the material, its degradation status, the chemical composition, and the density of traps (which stands for the quality of the material) monitoring the recombination decay in the ns time window.^7,12,23–26^ The spectrum does not undergo renormalization in the linewidth and peak position over time, because with our system, we are probing recombination mechanisms of cold charge carriers, been the hot-carriers recombination happening on the femtosecond timescale. The fluorescence decay associated spectra (DAS) were extracted with a home-built global analysis software and then fitted with a customized fitting software developed in LabView for extraction of the decay time. The fitting software is able to perform single, double, or three-exponential decay fit depending on the specific material investigated. It is worth to mention that the possibility to choose between the different types of exponential decay is essential for the proper analysis of the dynamics occurring in the materials/devices, being related to the fluence of the source exciting the sample. The data shown in Fig. 2 are in perfect agreement with observed data, supporting the validity and the quality of our method.^4,23^ Moreover, the investigated PL spectrum of the sample shows a peak around 764 nm, as the spectra previously reported in the literature for device with the same active material and architecture.^4,27^ In addition, from the fitting procedure (see Table S1 in the supplementary material) using a double-exponential decay, we have extrapolated the decay parameters. We adopted a function of the form: $y=A_{1}e^{-\left(x/\tau_{1}\right)}+A_{2}e^{-\left(x/\tau_{2}\right)}$, where $A_{1}$ and $A_{2}$ are the normalized amplitude of each decay component and $\tau_{1}$ and $\tau_{2}$ are the time constants, also referred to as fast and slow decay time life, respectively. Comparing the results in Table S1 with the parameters previously reported for the same kind of device, it is clear that the values are in the same range for both time constants and relative amplitudes. To go deeper in the comprehension of the recombination pathways as well as the ratio between the radiative and non-radiative pathways, the PLQY should be concomitantly employed. Importantly, the PLQY is related to the quasi-Fermi level splitting (QFLS) through the Shockley Queisser model allowing the quantification of the losses and the identification of the origin of the non-radiative recombination, directly relating these findings to the device parameters such as the solar cell open circuit voltage (V_OC_).^10,28^ As we mentioned before, PLQY measurements are performed by recording the spectra of the laser exciting sample and the emission spectrum of the sample itself in specific experimental conditions. In more detail, due to the expression of the PLQY—the ratio between the emitted photons over the absorbed ones—the collection of laser spectra with and without the sample in the integrating sphere is demanded in order to calculate the number of photons absorbed. Figure 3(a) shows an example of PLQY measurements on a perovskite thin film excited by a 520 nm laser. Two different wavelength regions are depicted: the short-wavelength region represents the absorption spectrum (black line) of the laser (calculated by the difference between laser spectra collected without and with the sample) while the long-wavelength region is related to the emission spectra of the sample (red line, with a 3× magnification). The system was calibrated by using a calibrated halogen lamp (LabSphere, MPN: SCL-1400) with specified spectral irradiance, which was shone into the integrating sphere. A spectral correction factor was established to match the spectral output of the detector to the calibrated spectral irradiance of the lamp. The spectral photon density was obtained from the corrected detector signal (spectral irradiance) by division through the photon energy, and the photon numbers of the excitation and emission obtained from numerical integration. By integrating absorption and emission spectra, it is possible to retrieve the number of photons emitted and absorbed and by their ratio to calculate the PLQY, in this case equal to 12%, in fair agreement with a high quality perovskite thin film.^29^ Beyond thin film (and solution) standard analyses, our system also enables the concomitant electrical measurements on working devices, leading to a complete characterization from the material to device. This is obtained by measuring, for instance, the EL spectrum of a solar cell [as shown in Fig. 3(b)], where the sample is sourced with a positive voltage and the emission is collected. In this case, the EL signal of the perovskite device is measured, enabling us to quantify the potential losses in the device interfaces and then provide a guideline for its further optimization. Altogether, the presented set of measurements allows for deriving a complete set of information retrieving the overall picture of the phenomena happening inside the material/device.^10,27^

## IV. CONCLUSIONS

In conclusion, we have presented a custom built compact, versatile, and low-cost system for the combined measurement of the time and wavelength resolved PL map, PLQY, and EL. Such a system combines good spectral and time resolution to high sensitivity, compactness, and low cost (around 60k EUR for the overall setup). With respect to competing solutions, our system offers the advantages of multiple measurements with the same robust and compact unit, a fast acquisition time, and an easy “plug and play” approach, which can be a valid alternative in the future market of electro-optical spectroscopic tools.

## SUPPLEMENTARY MATERIAL

See the supplementary material for additional data and discussion on the experimental setup.

## Data Availability

The data that support the findings of this study are available from the corresponding author upon reasonable request.
